# Supplementary material for: Saccharomyces boulardii (CNCM I-745) improves intestinal damage in sepsis by remodeling intestinal flora structure
Source: Front Cell Infect Microbiol. 2025 Jul 31;15:1602792. doi: 10.3389/fcimb.2025.1602792 (PMC12350329; doi:10.3389/fcimb.2025.1602792)
Supplement: Supplementary file 1 [file DataSheet1.zip › summary/summary/6_Taxonomy_community/1_Taxonomy_profiling/4_Order/Group/Order_abund_top30_Group.heatmap.pdf]

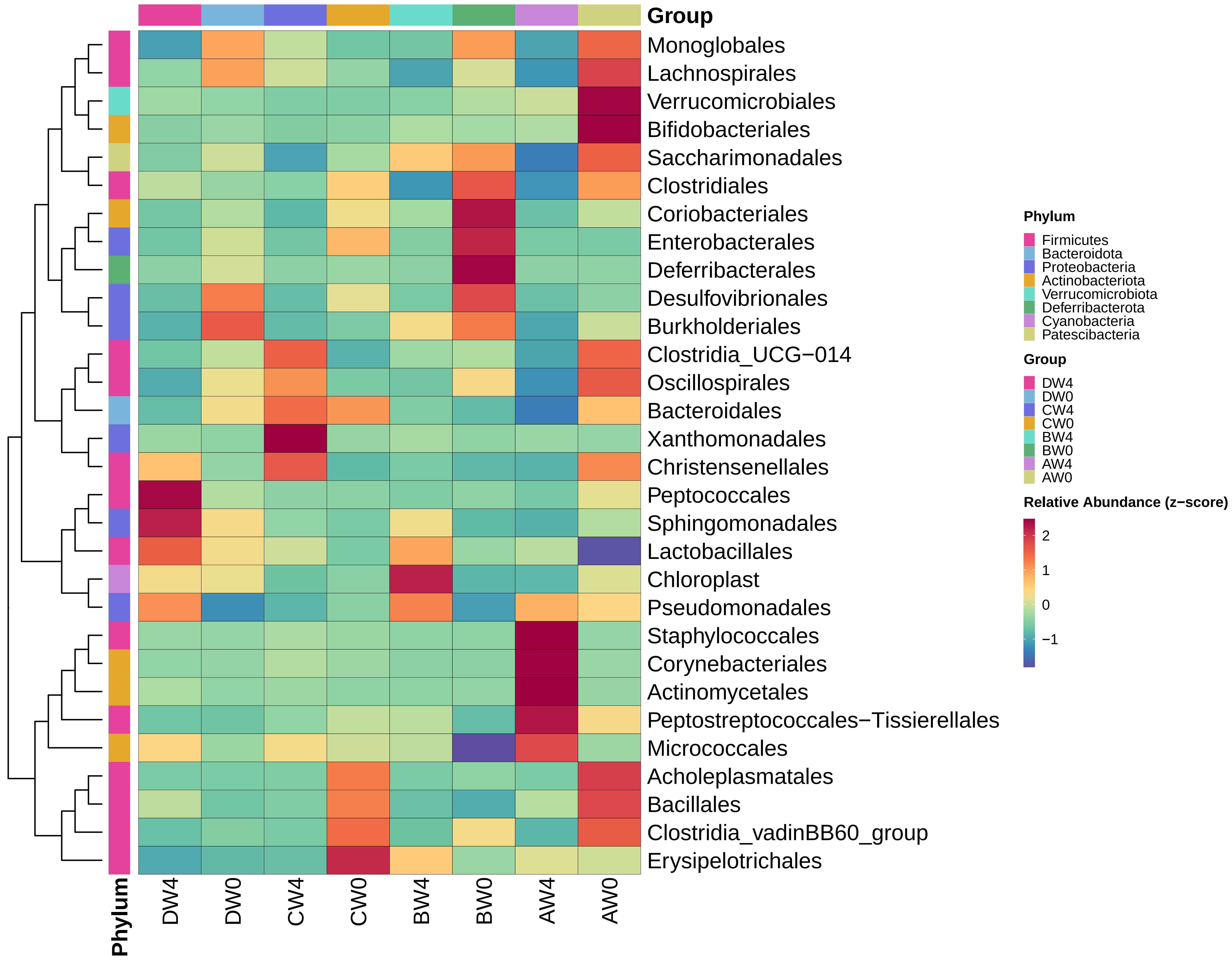
Group
Monoglobales
Lachnospirales
Verrucomicrobiales
Bifidobacteriales
Saccharimonadales
Clostridiales
Coriobacteriales
Enterobacterales
Deferribacterales
Desulfovibrionales
Burkholderiales
Clostridia_UCG−014
Oscillospirales
Bacteroidales
Xanthomonadales
Christensenellales
Peptococcales
Sphingomonadales
Lactobacillales
Chloroplast
Pseudomonadales
Staphylococcales
Corynebacteriales
Actinomycetales
Peptostreptococcales−Tissierellales
Micrococcales
Acholeplasmatales
Bacillales
Clostridia_vadinBB60_group
Erysipelotrichales
Phylum
DW4
DW0
CW4
CW0
BW4
BW0
AW4
AW0
Phylum
Firmicutes
Bacteroidota
Proteobacteria
Actinobacteriota
Verrucomicrobiota
Deferribacterota
Cyanobacteria
Patescibacteria
Group
DW4
DW0
CW4
CW0
BW4
BW0
AW4
AW0
Relative Abundance (z−score)
2
1
0
−1
